# Cyclodextrin-Based Strategies for Brain Drug Delivery: Mechanistic Insights into Blood–Brain Barrier Transport and Therapeutic Applications

**DOI:** 10.3390/pharmaceutics18040451

**Published:** 2026-04-07

**Authors:** Pirscoveanu Denisa Floriana Vasilica, Pluta Ion Dorin, Carmen Vladulescu, Cristina Popescu, Diana-Maria Trasca, Kristina Radivojevic, Renata Maria Varut, Ștefănița Bianca Vintilescu, Mioara Desdemona Stepan, George Alin Stoica

**Affiliations:** 1 Department of Neurology, Faculty of Medicine, University of Medicine and Pharmacy of Craiova, 200349 Craiova, Romania; denisa.pirscoveanu@umfcv.ro; 2 Faculty of Medical and Behavioral Sciences, Constantin Brâncuși University of Târgu Jiu, 210185 Târgu Jiu, Romania; dorin.pluta@e-ucb.ro; 3 Department of Biology and Environmental Engineering, Faculty of Horticulture, 200585 Craiova, Romania; carmen.vladulescu@edu.ucv.ro; 4 Department of Anatomy, ENT Doctor Department of Anatomy, University of Medicine and Pharmacy, 200349 Craiova, Romania; cristina.popescu@umfcv.ro; 5 Department of Internal Medicine, University of Medicine and Pharmacy of Craiova, 200349 Craiova, Romania; 6 Research Methodology Department, Faculty of Pharmacy, University of Medicine and Pharmacy of Craiova, 200349 Craiova, Romania; kristinaradivojevic03@gmail.com; 7 Department of Mother and Baby, University of Medicine and Pharmacy of Craiova, 200349 Craiova, Romania; bianca.vintilescu@umfcv.ro (Ș.B.V.); desdemona.stepan@umfcv.ro (M.D.S.); 8 Department of Pediatric Surgery, Faculty of Medicine, University of Medicine and Pharmacy of Craiova, 200349 Craiova, Romania; alin.stoica@umfcv.ro

**Keywords:** cyclodextrins, blood–brain barrier, brain-targeted drug delivery, receptor-mediated transcytosis, nanoparticles, neurodegenerative diseases

## Abstract

Cyclodextrins (CDs) have gained increasing attention as versatile platforms for enhancing drug delivery to the central nervous system, particularly in overcoming the restrictive properties of the blood–brain barrier (BBB). Owing to their unique cyclic oligosaccharide structure, CDs are capable of forming inclusion complexes with a wide range of therapeutic agents, thereby improving their solubility, stability, and bioavailability. In addition to their role as excipients, growing evidence indicates that CDs can actively modulate biological processes, including membrane fluidity and cholesterol homeostasis, which are critical factors in neurological disorders. This review explores the application of CDs in facilitating drug transport across the BBB through multiple mechanisms, including carrier-mediated transport, receptor-mediated transcytosis, and nanoparticle-based delivery systems. Special emphasis is placed on their use in the treatment of neurodegenerative and neurological diseases, such as Alzheimer’s disease, Parkinson’s disease, multiple sclerosis, Niemann–Pick type C disease, and other central nervous system disorders. In these contexts, CD-based formulations have demonstrated the ability to enhance brain targeting, reduce pathological protein aggregation, and improve therapeutic outcomes in preclinical models. This review uniquely integrates cyclodextrin’s physicochemical properties with specific blood–brain barrier transport mechanisms, proposing a structure–transport–therapy framework that enables a more predictive understanding of brain-targeted drug delivery.

## 1. Introduction

CDs are a family of cyclic macromolecules composed of D-glucopyranose units connected through α-1,4 glycosidic linkages, generated via enzymatic degradation of starch [1]. Their three-dimensional configuration resembles a truncated cone, characterized by a relatively lipophilic inner cavity and a hydrophilic outer surface. This spatial arrangement enables them to encapsulate a broad spectrum of hydrophobic molecules within their central cavity. The principal naturally occurring cyclodextrins—α-CD, β-CD, and γ-CD—contain six, seven, and eight glucose units, respectively, and are most frequently utilized in pharmaceutical applications owing to their suitable physicochemical characteristics and established safety profiles.

These cyclic oligosaccharides are widely employed as excipients in drug delivery systems, largely because of their structural versatility. The presence of reactive hydroxyl groups allows chemical modification, while their aqueous solubility and capacity to generate stable host–guest inclusion complexes with diverse compounds make them particularly valuable in formulation development [2,3,4]. Cyclodextrins are incorporated into numerous marketed medicinal products, and their safety and functional roles are acknowledged in major pharmacopeias and by regulatory authorities worldwide.

More recently, scientific focus has shifted beyond traditional inclusion complexes toward the development of sophisticated CD-based nanostructured systems. Such platforms can enhance therapeutic performance by improving the solubility and bioavailability of poorly water-soluble drugs, enabling more selective tissue distribution, extending systemic circulation time, and promoting synergistic effects between co-administered agents [5,6]. In addition to these delivery-related advantages, cyclodextrins demonstrate multifunctional properties: they may mitigate local irritation in gastrointestinal or ophthalmic formulations, enhance taste masking in oral preparations, prevent physicochemical incompatibilities among formulation components, and protect labile or volatile substances from degradation [7].

Despite their well-established role in enhancing drug solubility and stability, the true translational potential of cyclodextrins becomes particularly evident in the context of central nervous system therapy, where the restrictive nature of the blood–brain barrier represents a major obstacle to effective drug delivery.

The BBB represents one of the brain’s most important defense systems, preserving neural homeostasis by limiting exposure to potentially harmful circulating substances. While this protective role is essential for maintaining central nervous system (CNS) integrity, it simultaneously restricts the entry of most therapeutic agents [8]. A large proportion of small-molecule drugs, as well as biologics including peptides, proteins, and nucleic acid-based therapies, are unable to efficiently penetrate the BBB from the systemic circulation, thereby constraining pharmacological management of CNS diseases [9]. Although substantial progress has been achieved in targeted drug delivery technologies, achieving therapeutically relevant concentrations within brain tissue continues to be a major obstacle. Consequently, the design of advanced delivery platforms capable of overcoming or strategically navigating the BBB remains a central objective in CNS drug development.

Structurally and functionally, the BBB is characterized by tightly regulated permeability resulting from coordinated interactions between specialized cells and molecular components. Endothelial cells lining cerebral microvessels are interconnected by tight junction complexes composed of specific junctional proteins, which minimize uncontrolled paracellular diffusion [10]. In parallel, an array of membrane-bound receptors, carrier proteins, and ion channels modulate selective transcellular transport of nutrients, metabolites, and signaling molecules across the endothelial layer [11,12]. The barrier’s stability is further reinforced by supporting cells within the neurovascular unit. Astrocytes and pericytes play pivotal roles in maintaining BBB architecture and function; notably, pericytes influence permeability through the secretion of extracellular matrix components such as vitronectin, which interact with integrin receptors on endothelial cells [13].

**Figure 1:**
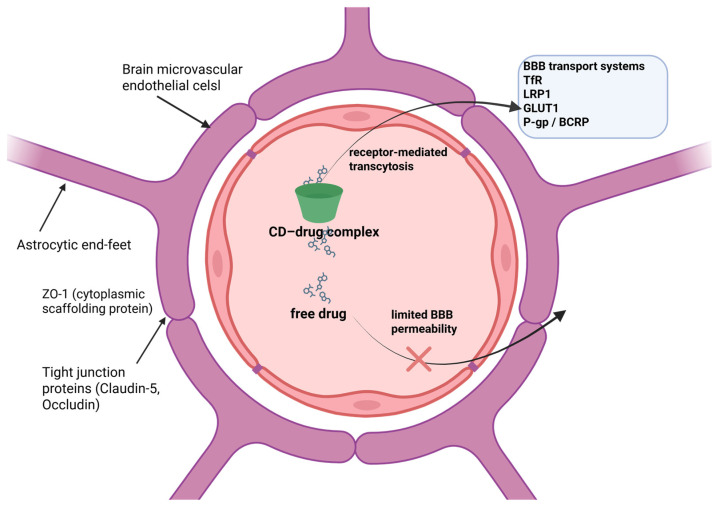
Structural organization of the blood–brain barrier and cyclodextrin-mediated drug transport. Tight junction proteins, including claudins, occludin, and ZO-1, are indicated at the intercellular junctions between brain microvascular endothelial cells. Created in BioRender. Varut, M. (2026); https://app.biorender.com/i-69a2fd2a88d58e73cbbb003b-blood-brain-barrier-transverse (accessed on 1 February 2026).

An additional layer of restriction is imposed by active efflux systems, including ATP-binding cassette (ABC) transporters such as ABCB1 (P-glycoprotein) and ABCG2 (Breast Cancer Resistance Protein, BCRP), which actively expel many xenobiotics back into the bloodstream [14]. Collectively, these anatomical and molecular features not only explain the limited brain uptake of numerous drugs but also provide mechanistic opportunities. In this context, cyclodextrins may be strategically engineered to take advantage of receptor-mediated pathways, carrier-dependent transport mechanisms, or modulation of tight junction dynamics to enhance CNS drug delivery. Figure 1 schematically illustrates the structural organization of the BBB and the principal mechanisms involved in cyclodextrin-mediated drug transport across the endothelial layer.

Efforts to enhance the therapeutic performance of CDs are increasingly directed toward the optimization of CD-based drug delivery systems (DDSs). Current research aims to improve site-specific accumulation of therapeutics while minimizing systemic toxicity and unwanted side effects [15,16]. Strategies to achieve this typically involve functionalization with targeting ligands or the formulation of CDs into nanoscale delivery platforms designed to promote selective tissue distribution and controlled drug release.

Several review articles have examined CD-based DDSs in distinct pathological settings. For example, Xing et al. [17,18] presented an extensive analysis of cyclodextrin-enabled delivery approaches in neurodegenerative conditions, including Alzheimer’s and Parkinson’s diseases. Their review underscored the adaptable chemical structure of CDs and their capacity to interact with biological barriers; however, it did not focus on the unique obstacles posed by glioblastoma (GB), nor on the specific therapeutic considerations required to address its marked heterogeneity and infiltrative behavior.

In a comparable manner, Păduraru et al. [19] discussed the application of CD-mediated delivery systems in oncology, emphasizing their favorable physicochemical attributes, biocompatibility, and versatility across multiple cancer types.

In contrast to previously published reviews that have primarily concentrated on either neurodegenerative disorders or oncological applications, the present work aims to provide a broader and integrative perspective on cyclodextrin-based strategies in the context of blood–brain barrier-associated drug delivery. Specifically, this review examines the role of cyclodextrins not only in Alzheimer’s and Parkinson’s diseases but also in brain malignancies and other CNS-related conditions where BBB permeability represents a critical therapeutic limitation. The selection of neurodegenerative diseases, glioblastoma, and lysosomal storage disorders was based on their strong dependence on blood–brain barrier permeability and the availability of mechanistic evidence regarding cyclodextrin-based delivery strategies. These disease categories represent distinct pathological contexts, protein aggregation, malignant proliferation, and metabolic dysfunction, thereby enabling a comparative evaluation of structure–transport–therapy relationships By bringing together insights from both neurodegenerative and oncological research, we seek to outline the current landscape of cyclodextrin-enabled approaches, highlight emerging trends, and identify key translational challenges that may shape future developments in this field. This review differs from previously published reports by going beyond descriptive analyses of cyclodextrin-based drug delivery systems and establishing a mechanistic framework that directly links cyclodextrin physicochemical properties to specific biological pathways involved in blood–brain barrier transport. While prior reviews have primarily focused on formulation aspects or disease-specific applications, the present work integrates these perspectives and comparatively analyzes cyclodextrin-based delivery systems across major central nervous system disorders, including neurodegenerative diseases, lysosomal storage disorders, and brain tumors. Particular emphasis is placed on the structure–transport–therapy relationship, highlighting how cavity size, derivatization, and carrier design influence cellular uptake mechanisms and brain exposure. Finally, the translational potential and current clinical limitations of cyclodextrin platforms are critically discussed in order to outline future directions for their implementation in brain-targeted therapies.

## 2. BBB—Structure and Targeted Delivery Strategies

The BBB represents a highly specialized interface that functionally separates the systemic circulation from the neural parenchyma. Structurally, it is formed by a coordinated assembly of brain microvascular endothelial cells, astrocytes, pericytes, the basement membrane, and intercellular junctional complexes, including tight junctions and adherens junctions (AJs) [20].

Brain microvascular endothelial cells (BMECs) are the principal cellular element of the BBB. These cells are sealed together by tight junction complexes composed of integral membrane proteins such as occludin and claudins (e.g., Claudin-3 and Claudin-5) [21], which are anchored intracellularly by scaffolding proteins including Zona Occludens-1, -2, and -3 (ZO-1, ZO-2, ZO-3) [22]. The organization of these junctional proteins confers marked selectivity to the BBB, substantially restricting paracellular diffusion of hydrophilic molecules and high-molecular-weight substances [23].

In addition to their junctional specialization, BMECs exhibit distinctive phenotypic features compared with peripheral endothelial cells, including the absence of fenestrations, minimal levels of nonspecific vesicular transport, and very limited passive paracellular permeability [24]. At the same time, they express a broad repertoire of membrane-bound receptors and transport systems that enable the regulated uptake and efflux of essential nutrients, metabolites, and signaling molecules across the barrier [25].

The basement membrane, synthesized by both endothelial cells and astrocytes, constitutes another key structural component of the BBB. It is primarily composed of collagen type IV, multiple laminin isoforms (laminin-1, -2, -4, and -5), and various glycoproteins [26], forming a supportive matrix that preserves barrier stability and organization. Astrocytes contribute further to BBB function through their perivascular end-feet, which closely envelop the microvasculature. By releasing diverse signaling mediators, astrocytes influence endothelial permeability and transport characteristics [27].

Pericytes, embedded within and surrounding the endothelial basement membrane, are equally essential. Together with BMECs and astrocytes, they form the neurovascular unit and actively participate in BBB development, maintenance, and dynamic regulation [28]. Moreover, enzymatic modulators such as matrix metalloproteinases and their endogenous inhibitors, including tissue inhibitors of metalloproteinase-1, have been shown to play important roles in controlling BBB integrity and remodeling processes [29].

Some receptors abundantly expressed at the BBB have been extensively investigated as gateways for receptor-mediated transcytosis, offering opportunities for brain-directed drug delivery. Rather than detailing individual nanomedicine formulations, the following overview highlights the principal receptor systems relevant to BBB transport and their therapeutic significance.

### 2.1. BBB—Receptors

#### 2.1.1. Transferrin Receptor (TfR1)

Transferrin receptor 1 (TfR1) is highly expressed on brain microvascular endothelial cells and is frequently upregulated in tumor cells, making it a prominent target for both neurological and oncological applications. Targeting strategies typically involve transferrin (Tf), anti-TfR antibodies, TfR-binding peptides, or other ligands with high affinity for the receptor. Upon ligand binding, TfR1 undergoes endocytosis, enabling receptor-mediated transcytosis across the BBB. This pathway has been widely explored for enhancing brain uptake of therapeutic agents [30,31,32]. Despite its advantages, Tf-based targeting may raise safety considerations, as transferrin-modified systems can potentially trigger immune recognition, which may affect therapeutic efficacy and tolerability.

#### 2.1.2. Low-Density Lipoprotein Receptor (LDLR) and LRP1

The low-density lipoprotein receptor (LDLR) is a transmembrane protein primarily involved in LDL internalization and cholesterol homeostasis. It is expressed at the BBB and recognizes ligands such as LDL and apolipoproteins. Ligands including Angiopep-2 and apolipoprotein E have been utilized to exploit LDLR-mediated uptake mechanisms for brain-targeted delivery [8]. Closely related to LDLR, low-density lipoprotein receptor-related protein 1 (LRP1) is a multifunctional receptor with a well-characterized expression profile at the BBB. LRP1 participates in physiological transport processes and has been investigated as a mediator of BBB transcytosis. Although LRP1-targeted strategies are promising, efficient translation requires precise control of ligand density and carrier design to optimize receptor engagement while minimizing rapid clearance or immune recognition [8].

#### 2.1.3. Lactoferrin Receptor (LfR)

LfR is expressed on brain endothelial cells and has also been detected in neurons associated with neurodegenerative conditions. Its involvement in receptor-mediated transport has positioned LfR as a candidate for CNS-directed delivery strategies [33].

#### 2.1.4. Melanotransferrin (MTf)

MTf is a surface-associated protein originally identified in melanoma cells and has been investigated as a targeting ligand in GB models. Antibody-mediated targeting of MTf has demonstrated the capacity to induce receptor-mediated transcytosis across the BBB and inhibit tumor cell proliferation in experimental settings [34].

#### 2.1.5. Insulin Receptor (IR)

The IR is abundantly expressed on brain microvascular endothelial cells and represents another pathway for receptor-mediated BBB transport. Insulin or insulin analog-based conjugates can exploit this system to facilitate brain entry. Experimental approaches using engineered insulin fusion proteins have demonstrated targeted delivery to specific neuronal populations, including hippocampal neurons, suggesting potential applications in neurodegenerative disorders such as Alzheimer’s disease [35].

#### 2.1.6. Folate Receptor (FR)

Members of the folate receptor family (FRα, FRβ, FRγ, FRδ) are encoded by FOLR1–4 genes. Among these, FRα is notably expressed at the BBB and in certain tumor tissues, supporting its consideration as a dual target in CNS and oncological contexts [36].

#### 2.1.7. Nicotinic Acetylcholine Receptor (nAChR)

nAChRs are broadly distributed in brain tissue, including endothelial cells of cerebral capillaries. Peptide ligands such as RVG29 exhibit affinity for nAChRs and have been employed to enhance BBB penetration in experimental models of Alzheimer’s disease [37].

#### 2.1.8. Additional Receptors

Other receptor systems expressed at the BBB include scavenger receptor class B type I (SR-BI), diphtheria toxin receptor (DTR), and bradykinin B2 receptor (B2R). SR-BI interacts primarily with high-density lipoproteins and may support ligand-mediated brain delivery [38]. DTR can be utilized experimentally for targeted cellular uptake through toxin- or derivative-based conjugation strategies [39]. B2R, a G protein-coupled receptor activated by bradykinin, has been implicated in modulating BBB permeability and facilitating transport processes [40]. Among the receptors described, TfR remains one of the most extensively investigated targets due to its high expression at the BBB and well-characterized transcytosis mechanisms, although its translational application may be limited by potential immunogenicity and receptor saturation. LRP1- and LDLR-based systems offer promising alternatives with broader ligand versatility, but require careful optimization of ligand density and carrier design. The insulin receptor exhibits high transcytotic efficiency; however, its physiological role raises safety concerns related to metabolic interference, while other receptors such as LfR and FR remain less advanced in clinical translation. From a translational perspective, TfR- and IR-based systems represent the most advanced approaches, with several strategies progressing into clinical or early clinical development. In contrast, receptors including LRP1, LDLR, folate receptor, and nicotinic acetylcholine receptor remain predominantly at the preclinical stage. Overall, the selection of a specific receptor depends on balancing transport efficiency, targeting specificity, and safety considerations.

### 2.2. BBB—Transporter-Mediated and Tight Junction Protein Mediated Transportation

Transporter-mediated and tight junction protein-mediated transportation has been summarized in Table 1 and Table 2.

**Figure 2:**
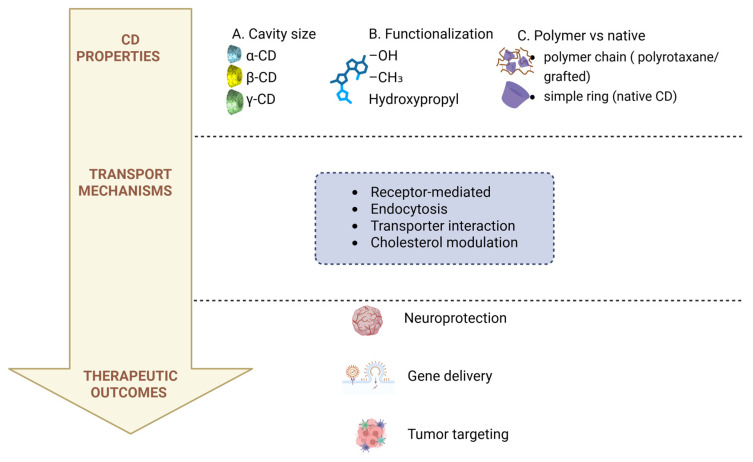
Mechanistic framework illustrating the structure–transport–therapy relationship in cyclodextrin-based brain drug delivery. Cyclodextrin physicochemical properties, including cavity size, functionalization, and polymeric architecture, influence transport mechanisms such as receptor-mediated transcytosis, endocytosis, transporter interaction, and cholesterol modulation, ultimately determining therapeutic outcomes including neuroprotection, gene delivery, and tumor targeting. Created in BioRender. Varut, M. (2026); https://app.biorender.com/i-69c1a80fb9a77470352f4a86-untitled (accessed on 4 February 2026).

To provide a conceptual overview of the structure–transport–therapy relationship underlying cyclodextrin-based brain delivery systems, the key physicochemical properties, transport mechanisms, and therapeutic outcomes are summarized in Figure 2.

Although cyclodextrins do not directly act as substrates for most BBB transporters, they can indirectly facilitate transporter-mediated drug delivery. By forming inclusion complexes, cyclodextrins enhance drug solubility and maintain therapeutically relevant concentrations of the free drug fraction available for transporter recognition. In addition, cyclodextrins can modulate membrane fluidity and cholesterol content, which may influence the activity of transport proteins such as efflux transporters. Furthermore, cyclodextrin-based nanocarriers can be functionalized with ligands targeting specific transporter systems, thereby enabling selective uptake across the BBB. These combined effects highlight the potential of cyclodextrins to support, rather than directly mediate, transporter-based delivery mechanisms. However, in the case of receptor-mediated targeting strategies, an important limitation arises from the widespread expression of certain receptors, such as the insulin receptor and folate receptor, in peripheral tissues including the liver and kidneys. This may lead to off-target drug accumulation and reduced brain specificity. Therefore, the design of targeted cyclodextrin-based systems must consider ligand affinity, dosing strategies, and carrier optimization to enhance BBB selectivity while minimizing systemic distribution.

## 3. CDs

### 3.1. ADMET Analysis of CDs

β-amylases, which cleave starch from the non-reducing termini of carbohydrate chains, exhibit resistance toward CDs. In contrast, α-amylases, acting endolytically within the polysaccharide chain, are capable of progressively degrading starch. Human α-amylase is present in several physiological fluids, including saliva, tear fluid, and bile, where it efficiently hydrolyzes linear dextrins. However, the cyclic architecture of CDs, together with their chemical substitutions, limits enzymatic access and prevents effective hydrolysis. Among native CDs, unsubstituted γ-CD remains the most susceptible to α-amylase-mediated degradation [56,57,58,59].

The formation of inclusion complexes further restricts enzymatic interaction, thereby reducing the hydrolysis rate of CDs and significantly slowing the degradation of substituted γ-CD derivatives. CDs that are resistant to enzymatic breakdown in the upper gastrointestinal tract undergo bacterial fermentation in the distal intestine [60]. Orally administered γ-CD is almost completely metabolized within the gastrointestinal tract, while β-CD and γ-CD derivatives are primarily degraded by colonic microbiota. Compared to β-CD, α-CD demonstrates a slower absorption profile.

Following intravenous administration, approximately 90% of CDs are eliminated unchanged via renal glomerular filtration, while the remaining fraction undergoes alternative clearance pathways, including biliary excretion and hepatic metabolism [61,62]. Clinical data indicate that sugammadex, hydroxypropyl-β-cyclodextrin (HP-β-CD), and SBE-β-CD are predominantly excreted unchanged through renal filtration [63,64,65]. These compounds exhibit comparable pharmacokinetic profiles, with elimination half-lives ranging between 1.6 and 1.9 h. The relatively short systemic half-life of cyclodextrins may limit sustained drug exposure at the level of the central nervous system, particularly in conditions requiring prolonged therapeutic concentrations. Rapid renal elimination reduces systemic circulation time, which can restrict the duration of drug availability for blood–brain barrier transport. Consequently, effective CNS delivery may require repeated dosing regimens or the development of modified-release formulations and cyclodextrin-based nanocarriers designed to prolong systemic exposure and enhance brain uptake. More than 90% of administered CDs are cleared within 6 h after parenteral administration, and over 99.9% are eliminated within 24 h. Consequently, accumulation is not expected in individuals with normal renal function, even at elevated doses [64]. In contrast, patients with severe renal impairment (creatinine clearance <10 mL/min) may exhibit CD accumulation. Moreover, in patients with advanced hepatic dysfunction, including acute-on-chronic liver failure, systemic pharmacokinetics may be profoundly altered due to impaired metabolic capacity and multiorgan interactions, which should be carefully considered when administering cyclodextrin-based formulations [66].

In the gastrointestinal tract, bacterial metabolism of CDs leads to the formation of oligosaccharides, monosaccharides, and gaseous byproducts such as hydrogen, carbon dioxide, and methane [67]. While CDs do not accumulate in individuals with normal elimination capacity, accumulation may occur in those with impaired renal function. The oral bioavailability of HP-β-CD is low (approximately 0.5–3.3%), with a substantial proportion undergoing colonic degradation and 50–65% being excreted unchanged in feces [59].

Most highly water-soluble and hydrophilic CDs share similar pharmacokinetic characteristics [59]. Notable differences include variations in small intestinal degradation and the relatively higher oral bioavailability of RMCD (up to 12%), which is associated with its stronger binding affinity and extended half-life (up to 7 h).

The safety and toxicity profiles of CDs used in pharmaceutical formulations have been extensively evaluated [68,69,70,71,72]. Due to their hydrophilic nature, low octanol–water partition coefficients, and high capacity for hydrogen bonding, CDs exhibit limited ability to passively diffuse across biological membranes [73,74,75,76]. Transporter-mediated uptake has been suggested but remains minimal, contributing to their generally poor oral bioavailability. For example, RM-β-CD demonstrates an oral bioavailability of approximately 12% in animal models (log K_o_/*w* ≈ 6) [77]. Native CDs (α-, β-, γ-CD) and their hydrophilic derivatives are considered to have low toxicity when administered orally, largely due to restricted systemic absorption [73,78,79]. From a brain delivery perspective, these pharmacokinetic characteristics are particularly relevant. The limited passive diffusion of most therapeutic agents across the blood–brain barrier requires the use of carrier systems capable of maintaining sufficient plasma concentrations while minimizing systemic toxicity. The rapid renal elimination of certain cyclodextrin derivatives also has important implications for dosing strategies in central nervous system disorders, where sustained drug exposure is often required. The pharmacokinetic properties of cyclodextrins have important implications for their use in CNS drug delivery. Their limited passive diffusion across biological membranes, including the blood–brain barrier, prevents significant intrinsic brain penetration, supporting their role as carrier systems rather than active therapeutic agents. At the same time, their rapid renal elimination and low systemic accumulation contribute to a favorable safety profile, which is essential for repeated administration in chronic neurological conditions. Furthermore, their ability to enhance drug solubility and maintain adequate plasma concentrations facilitates the sustained availability of the active compound for BBB transport. These characteristics collectively support the use of cyclodextrins as safe and effective platforms for brain-targeted drug delivery.

### 3.2. The Effect of CDs on Drugs’ Solubility

**Figure 3:**
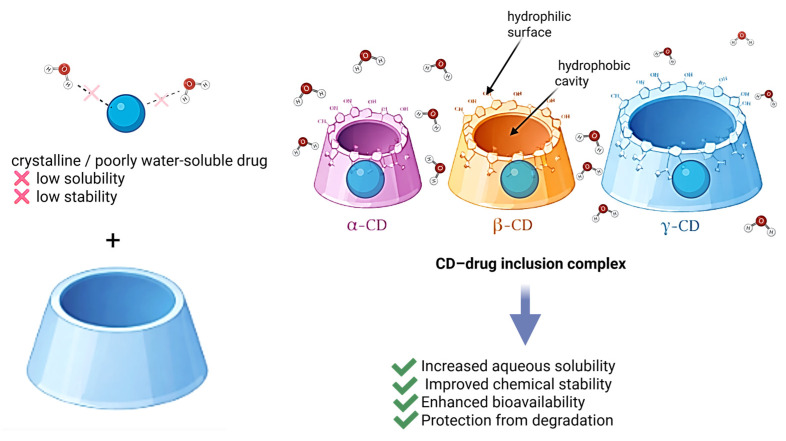
Schematic representation of cyclodextrin–drug inclusion complex formation and its pharmaceutical advantages. Created in BioRender. Varut, M. (2026); https://BioRender.com/ac7091r (accessed on 1 February 2026).

Cyclodextrins play a critical role in improving the delivery of poorly water-soluble drugs. Their primary mechanism involves enhancing apparent solubility and dissolution rates through inclusion complex formation or solid dispersion systems. Additionally, CDs can function as hydrophilic carriers for drugs that do not readily form inclusion complexes, or they may enhance the dissolution of high-dose drugs such as paracetamol, where complex formation is limited [69]. The apparent stability constant (K, M^−1^) of CD–drug complexes varies widely, typically ranging from 0 to 100,000. Cyclodextrins enhance the apparent solubility and dissolution rate of poorly water-soluble drugs primarily through inclusion complex formation (Figure 3).

Among available derivatives, methylated CDs with low degrees of substitution are considered particularly effective solubilizers. Improved solubility is also associated with reduced drug crystallinity when dispersed or complexed with CDs. Furthermore, CDs can enhance drug solubility without requiring solid-state complex formation by generating inclusion complexes directly in the dissolution medium [75].

SBE-β-CD has demonstrated strong solubilizing capacity for a wide range of pharmaceutical compounds, outperforming native β-CD, although it remains less effective than DM-β-CD. CDs can also act as release-modifying agents; for instance, β-CD has been shown to enhance the release of poorly soluble drugs such as naproxen and ketoprofen from polymer-based matrices, including hydrophilic swellable systems like hydroxypropyl methylcellulose (HPMC). Similarly, β-CD improves the dissolution behavior of theophylline from HPMC matrices by increasing both apparent solubility and permeability [80].

A key determinant of oral drug absorption is aqueous solubility, which directly influences bioavailability. Enhancing the solubility and dissolution rate of drugs, particularly those classified under BCS classes II and IV, can significantly improve their absorption profiles. Cyclodextrins facilitate this process through the formation of non-covalent inclusion complexes in solution, thereby increasing the apparent solubility of therapeutic agents.

### 3.3. Role of CD Complexation in Enhancing Drug Stability

Pharmaceutical stability represents a critical quality attribute of medicinal products. Beyond its direct impact on therapeutic efficacy and patient safety, it also underpins decisions related to formulation design, manufacturing processes, packaging selection, as well as storage and distribution conditions [81]. The concept of pharmaceutical stability refers to the ability of a drug product to maintain its identity, potency, and purity over time [82].

Compounds containing structurally flexible functional groups may undergo chemical degradation under specific environmental conditions. Several degradation pathways have been identified, including hydrolysis, dehydration, isomerization, racemization, oxidation, and photodegradation. In addition to chemical instability, drugs may also experience physical instability, leading to alterations in physicochemical properties. Phase transitions are among the factors that can contribute to such physical changes. A considerable number of therapeutically effective active pharmaceutical ingredients exhibit limited clinical applicability due to inadequate chemical or physical stability. Within this context, strategies aimed at improving drug stability remain a significant focus in pharmaceutical research. One commonly employed approach involves the formation of complexes between drug molecules and excipients. Complexation is defined as a reversible interaction between a substrate and a receptor molecule, resulting in a system with distinct stoichiometric and physicochemical properties compared to the individual components. CD-based complexation relies on the encapsulation of labile drug molecules within the CD cavity, thereby providing a protective microenvironment that reduces exposure to degradation factors. This molecular shielding effect contributes to enhanced stability against various degradation pathways. Among CD derivatives, SBE-β-CD has been shown to significantly improve the stability of chemically unstable compounds relative to other CDs [83].

### 3.4. CDs—Natural Product Inclusion Complex

Cyclodextrins have also been extensively investigated for their ability to stabilize bioactive compounds derived from natural sources, including polyphenols, alkaloids, terpenoids, flavonoids, and essential oils. Numerous studies have demonstrated that CD complexation can enhance the in vitro biological activity of such compounds, particularly those with anticancer, anti-inflammatory, and neuroprotective properties.

Cutaneous malignant melanoma is characterized not only by aggressive tumor proliferation but also by a complex tumoral and peritumoral inflammatory microenvironment that significantly influences disease progression and therapeutic response [84]. For example, ursolic acid, a triterpenoid commonly found in medicinal plants and dietary sources, exhibited markedly improved antiproliferative activity against melanoma cell lines when complexed with HP-β-CD compared to its free form [85]. Similarly, inclusion complexes have enhanced the anticancer potential of other terpenoids, such as saikosaponin-d and betulinic acid. In particular, HP-β-CD improved both the solubility and anticancer efficacy of saikosaponin-d in human skin squamous cell carcinoma models [59,86], while β-CD-based encapsulation of betulinic acid inhibited the proliferation of human breast cancer cells [87].

Additional evidence highlights the role of CD systems in improving the biological activity of other natural compounds. Fucoxanthin, a marine-derived carotenoid, demonstrated enhanced anticancer activity in human colorectal carcinoma models when formulated with HP-β-CD [88]. Likewise, the stability of alkaloids such as camptothecin and luotonin A, derived from Camptotheca acuminata, was significantly improved through complexation with β-CD and HP-β-CD. These inclusion complexes also exhibited increased cytotoxic effects across multiple cancer cell lines compared to the free compounds [89].

Flavonoids have also benefited from CD complexation strategies. For instance, the complex formed between dihydroquercetin (taxifolin) and β-CD displayed enhanced antioxidant and anticancer activity in hepatocellular carcinoma models. Similarly, β-CD improved the cytotoxic properties of mansonone G, a naphthoquinone, in lung cancer cells. The enhanced biological performance of these compounds is largely attributed to improved stability and solubility resulting from inclusion complex formation [89,90].

By protecting bioactive molecules from degradation and increasing their aqueous solubility, CDs facilitate improved cellular uptake and bioavailability. Curcumin, a well-known curcuminoid with recognized anticancer potential, has been widely studied in CD-based delivery systems. Encapsulation within various CD formulations has been shown to enhance its solubility and, consequently, its antitumor activity. For example, curcumin-loaded HP-β-CD liposomal systems demonstrated increased cytotoxicity against osteosarcoma and breast cancer cell lines [90]. Furthermore, formulations combining curcumin with chitosan microspheres and HP-β-CD improved its therapeutic index in human colorectal cancer models [91].

Despite these promising findings, it is important to note that most studies evaluating the anticancer effects of CD-encapsulated bioactive compounds are limited to in vitro models. This raises uncertainties regarding their translational potential in vivo. Nevertheless, the available evidence supports the potential of CD-based systems to enhance the therapeutic performance of bioactive molecules, warranting further investigation in this field.

### 3.5. Types and Characteristics of CD Polymers

CD-based polymers represent a class of macromolecular systems in which CDs constitute the fundamental structural units, forming polymeric networks or architectures such as polyrotaxanes, polypseudorotaxanes, and grafted CD polymers [92].

Polyrotaxanes are supramolecular assemblies generated through the covalent linkage of rotaxane units, where cyclic CD molecules are threaded onto a linear polymer chain and stabilized by bulky end groups [93,94]. Within these systems, CDs serve as essential structural components, with α-CD/polyethylene oxide-based polyrotaxanes being among the most widely investigated [92]. As an example, Yu et al. developed supramolecular nanostructures using an amphiphilic diblock copolymer as the linear axis and amino-functionalized β-CD as the cyclic component, relying on host–guest interactions with poly(ε-caprolactone) segments to drive assembly [95].

Polypseudorotaxanes share a similar threaded architecture; however, they lack terminal capping groups, resulting in a more dynamic and reversible supramolecular structure. For instance, Crivello et al. reported a poly(pseudo)rotaxane-based hydrogel system formed from α-CD and tailored polyether polyurethanes, designed for the incorporation of lignin–cobalt nanoparticles, demonstrating potential for applications such as chronic wound treatment [96].

In addition to these architectures, CDs can be chemically grafted onto a wide variety of polymeric backbones, including linear, branched, cationic, anionic, and copolymeric systems. Among these, linear grafted CD polymers are the most extensively explored, with common examples including chitosan [97], alginate [98], and cholesterol-based systems [99]. Grafting strategies have enabled the development of stimuli-responsive delivery platforms. For example, Wang et al. utilized epichlorohydrin as a cross-linking agent to attach β-CD onto chitosan, resulting in a system capable of controlled drug release that responds to variations in pH and temperature, specifically for the anticancer agent etoposide [100]. Similarly, Khodayari et al. engineered a hybrid system by grafting hyaluronic acid/β-CD onto iron oxide nanoparticles for doxorubicin delivery. This formulation demonstrated enhanced drug release under acidic conditions (pH 5.6), achieving 92.43% release over 48 h, compared to 77.05% at physiological pH (7.4) [101]. A comparative perspective between native cyclodextrins and cyclodextrin-based polymers highlights important differences in their applicability and delivery efficiency. Native CDs are primarily used as solubilizing agents through inclusion complex formation, offering advantages such as well-established safety profiles, low toxicity, and ease of formulation. However, their intrinsic ability to cross the blood–brain barrier is limited, and their effects on drug delivery are often indirect, mainly by improving solubility and maintaining the free drug fraction available for transport. In contrast, cyclodextrin-based polymers, including polyrotaxanes and grafted systems, provide enhanced structural versatility and improved drug delivery performance. These systems enable higher drug loading capacity, increased stability, and the possibility of controlled or stimuli-responsive release. Moreover, polymeric CD platforms can be functionalized with targeting ligands, facilitating receptor-mediated transcytosis and improving BBB penetration. Their larger size and tunable surface properties also promote cellular uptake via endocytosis, which is particularly advantageous for the delivery of macromolecules such as peptides, proteins, and nucleic acids. However, these advantages are accompanied by increased complexity in synthesis, characterization, and regulatory approval, as well as potential concerns regarding long-term safety and biodegradability. Therefore, while native CDs remain suitable for simple solubilization strategies and small-molecule delivery, polymeric cyclodextrin systems represent more advanced platforms for targeted and efficient brain drug delivery, particularly in the context of nanomedicine and gene therapy applications.

### 3.6. Endocytosis of Cyclodextrins

In recent years, there has been increasing interest in the potential for CDs and their complexes to undergo endocytosis. This pathway is generally less significant for small, lipophilic molecules, which can readily cross barrier membranes. However, plasma membrane invagination and the formation of endocytic vesicles can expand the available surface for CD–membrane interactions, making this process relevant for drug absorption through the gastrointestinal tract. For larger molecules, such as peptides or oligonucleotides, endocytosis may represent a crucial route for crossing cellular membranes.

Endocytosis of fluorescently labeled β-cyclodextrin derivatives has been demonstrated in several cell types. Fluorescein- and rhodamine-labeled random methyl-β-cyclodextrin, fluorescein-labeled hydroxypropyl-β-cyclodextrin, and soluble β-cyclodextrin polymers were tested on human intestinal epithelial Caco-2 cells [102,103]. Fluorescein-labeled methyl-β-cyclodextrin was studied in HeLa cells [104], methyl-β-cyclodextrin–dextran–AlexaFluor546 polymers in NPC mutant human fibroblasts [105], and mono-4-(N-6-deoxy-6-amino-β-cyclodextrin)-7-nitrobenzofuran in HepG2 and SK-MEL-24 cells [106]. In Caco-2 cells, macropinocytosis was identified as the main uptake mechanism, whereas in HeLa cells, clathrin-mediated endocytosis predominated. These observations indicate that endocytic pathways may vary depending on the cell type, and multiple mechanisms can operate concurrently. Although systematic studies on the influence of fluorophore type or structure–activity relationships are lacking, endocytosis appears to occur across a range of fluorophore labels and CD derivatives.

It is important to distinguish receptor-mediated uptake of functionalized CDs from these nonspecific processes. For instance, folate-conjugated methyl-β-cyclodextrin [107] and mannose-modified β-cyclodextrin-based polyrotaxanes [108] undergo receptor-mediated endocytosis via interactions with folate and mannose receptors, respectively. A notable effect relevant to endocytosis involves membrane cholesterol extraction by methyl-β-cyclodextrins. Cell membranes are dynamic and asymmetric, with anionic phospholipids predominantly in the cytosolic leaflet, generating electrostatic repulsion and intrinsic curvature. Cholesterol in the membrane helps stabilize curvature by balancing repulsive forces and undergoing spontaneous flip-flop between leaflets. Removal of cholesterol by CDs increases negative surface charge density on the inner leaflet, promoting positive curvature and rapid membrane internalization [109]. In this way, CDs can act as nonspecific inducers of membrane internalization at biological barriers, facilitating uptake of surrounding fluid and potentially contributing to drug absorption. However, excessive depletion of membrane cholesterol can suppress endocytosis [110,111] while enhancing exocytosis [112]. While cholesterol depletion by cyclodextrins may facilitate membrane internalization and enhance drug uptake, it may also raise safety concerns, particularly in neuronal tissues where cholesterol plays a critical role in membrane stability, synaptic function, and signal transduction. Excessive or prolonged cholesterol extraction can disrupt membrane integrity, alter receptor function, and impair neuronal viability. Therefore, careful control of cyclodextrin type, concentration, and exposure duration is essential to balance enhanced delivery with potential neurotoxicity, especially in the context of repeated or long-term administration. Overall, CD–membrane interactions are highly complex and depend on the cyclodextrin type, concentration, and specific cellular context. The ability of cyclodextrins to enter cells through multiple endocytic pathways represents a key advantage for brain-targeted delivery. Clathrin-mediated endocytosis is typically associated with receptor-targeted systems, whereas caveolae-mediated uptake and macropinocytosis may facilitate the internalization of larger cyclodextrin-based assemblies. The predominance of a specific pathway depends on factors such as cyclodextrin derivative, particle size, and surface functionalization, which ultimately determine the efficiency of transcytosis across the brain endothelium. Importantly, no single uptake mechanism can be considered universally predominant for cyclodextrin-based nanocarriers crossing the blood–brain barrier. However, receptor-mediated transcytosis is generally regarded as the most efficient and translationally relevant mechanism, particularly for systems functionalized with specific targeting ligands, while other endocytic pathways contribute mainly to nonspecific cellular uptake. In this context, cholesterol extraction by cyclodextrins emerges as a key underlying mechanism that links membrane remodeling with both enhanced permeability and potential toxicity. By altering lipid raft organization and membrane fluidity, cholesterol depletion may facilitate BBB transport, but it also poses risks to neuronal membrane integrity and function. Therefore, this dual effect must be carefully considered when designing cyclodextrin-based delivery systems for CNS applications.

### 3.7. Bioinformatics and Computational Approaches in CD-Based Brain Delivery

Recent advances in bioinformatics and computational modeling have significantly contributed to the rational design of cyclodextrin-based drug delivery systems. Molecular docking and molecular dynamics simulations are widely used to investigate host–guest interactions between cyclodextrins and drug molecules, providing insights into binding affinity, stability, and inclusion complex formation. These approaches enable the prediction of optimal cyclodextrin derivatives and functionalization patterns for improved drug loading and release behavior. In addition, in silico ADMET prediction tools are increasingly employed to estimate pharmacokinetic properties, including solubility, permeability, blood–brain barrier penetration, and toxicity. Such methods facilitate early-stage screening of candidate systems, reducing the need for extensive experimental testing. Network-based and systems biology approaches have also been applied to identify relevant transport pathways and receptor targets involved in BBB translocation, supporting the design of ligand-functionalized cyclodextrin carriers. Furthermore, machine learning techniques are emerging as powerful tools for predicting structure–property relationships and optimizing formulation parameters.

Overall, bioinformatics-driven strategies provide a valuable framework for accelerating the development of cyclodextrin-based delivery platforms. While these approaches offer significant advantages in terms of efficiency and predictive capability, their accuracy remains dependent on model quality and experimental validation, highlighting the need for integrated computational–experimental workflows.

## 4. Brain-Delivered Drugs with CDs

**Figure 4:**
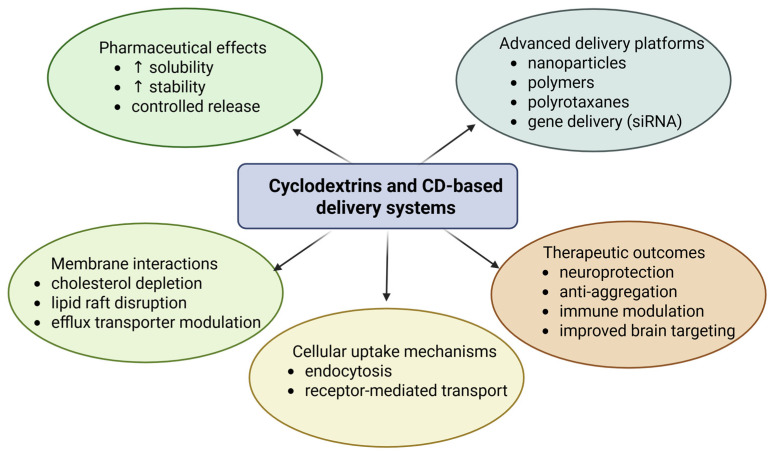
Multifunctional roles of cyclodextrins and CD-based delivery systems in brain-targeted therapy. Cyclodextrins enhance drug solubility and stability, modulate membrane cholesterol and efflux transporters, facilitate cellular uptake via endocytosis and receptor-mediated transport, and enable advanced nanocarrier and gene delivery platforms, ultimately improving therapeutic outcomes in central nervous system disorders. Created in BioRender. Varut, M. (2026); https://BioRender.com/m7cgip2 (accessed on 2 February 2026).

The delivery of pharmacologically active compounds to the brain remains a significant challenge due to the presence of highly specialized protective barriers. Although the brain receives substantial blood flow, the penetration of therapeutic agents is tightly restricted by two key interfaces, the BBB and the blood–cerebrospinal fluid barrier, both of which regulate the exchange of endogenous and exogenous substances [113]. The multiple mechanisms through which cyclodextrins enhance brain drug delivery and therapeutic efficacy are schematically illustrated in Figure 4.

Several studies have demonstrated the effectiveness of ligand–receptor interactions in enhancing the brain delivery of cyclodextrin-based systems. For example, lactoferrin-conjugated β-cyclodextrin nanocarriers have shown significantly improved BBB permeability due to interaction with lactoferrin receptors expressed on brain endothelial cells, resulting in increased brain accumulation of the therapeutic cargo. Similarly, transferrin receptor-targeted systems have been widely investigated to facilitate receptor-mediated transcytosis across the BBB, enabling improved transport of nanoparticles and drug–cyclodextrin complexes. In addition to receptor targeting, endocytosis also represents a major uptake pathway for cyclodextrin-based carriers. Polymeric cyclodextrin systems and amphiphilic CD nanoparticles have been shown to enter cells through clathrin-mediated endocytosis, caveolae-mediated uptake, or macropinocytosis, depending on particle size, surface charge, and functionalization. For instance, amphiphilic β-cyclodextrin carriers used for siRNA delivery in glioblastoma models have demonstrated efficient cellular uptake via macropinocytosis and clathrin-mediated endocytosis, leading to significant gene silencing effects. These examples highlight the importance of combining receptor-targeting ligands with endocytosis-driven uptake mechanisms to maximize the therapeutic efficiency of cyclodextrin-based brain delivery systems.

To access the brain parenchyma, compounds must first traverse the BBB, a complex structure formed by BMECs, astrocytes, pericytes, and microglia. BMECs exhibit distinctive features, including the absence of fenestrations, reduced vesicular transport, and the presence of tight intercellular junctions, all of which collectively limit the passive diffusion of molecules into the brain interstitial fluid. Beyond neurodegenerative disorders, alterations in systemic metabolic profiles, including disturbances in amino acid homeostasis observed in neurodevelopmental conditions such as autism spectrum disorder, further underscore the importance of understanding nutrient transport and metabolic regulation at the level of the blood–brain barrier [114].

Among targeting strategies, lactoferrin (Lf), a positively charged iron-binding glycoprotein belonging to the transferrin family, has been explored as a ligand for BBB transport. Conjugation of Lf to β-CD via a heterobifunctional polyethylene glycol linker (NHS-PEG-MAL) results in the formation of Lf-CD systems. These constructs have demonstrated enhanced brain delivery, as evidenced by increased BBB permeability and a significantly higher brain exposure (area under the curve) compared to non-targeted formulations [115].

Cyclodextrin-based prodrugs may exhibit limited aqueous solubility due to the presence of lipophilic moieties; however, derivatives such as HP-β-CD can improve both the solubility and chemical stability of incorporated compounds [116,117,118]. Consequently, formulation design plays a central role in optimizing cyclodextrin-based systems for brain targeting.

In vitro BBB models, such as co-cultures of bovine brain endothelial cells (BCECs) and astrocytes, have been widely used to evaluate cyclodextrin interactions with the barrier [119,120]. These models demonstrate high electrical resistance and low permeability, mimicking in vivo conditions. Studies assessing different CD types and derivatives indicate that native CDs can increase endothelial permeability in a concentration-dependent manner, with effects varying among α-, β-, and γ-CDs. Chemical modification influences toxicity, as hydroxypropylation generally reduces adverse effects for β- and γ-CDs, whereas methylation shows variable outcomes [113,121,122].

Despite their ability to interact with membrane components such as cholesterol and phospholipids, cyclodextrins exhibit limited intrinsic permeability across the BBB. Experimental data suggest that their transport across endothelial cells is minimal and comparable to that of efflux transporter substrates, a finding supported by both in vitro and in vivo studies [123,124].

Importantly, cyclodextrins can indirectly enhance drug delivery by modulating membrane properties and transporter activity. For example, certain derivatives, including RAMEB and CRYSMEB, have been shown to increase the transendothelial transport of drugs such as doxorubicin, likely through cholesterol depletion and disruption of lipid raft structures associated with efflux transporters [113,125]. This effect may also involve the inhibition of P-glycoprotein activity, thereby reducing drug efflux. In contrast, other derivatives, such as γ-CD and HP-γ-CD, demonstrate limited influence on drug transport [126,127].

Additionally, cyclodextrin-based nanocarriers, including quaternary ammonium β-CD systems and polymeric nanoparticles incorporating β-CD, have shown the ability to enhance drug transport across endothelial monolayers without compromising barrier integrity [127,128].

### 4.1. CDs with Receptors

Recent findings indicate that β-CD, a compound commonly used as an inert solubilizer, can exert notable effects on the kinetics of GABAa receptor conformational transitions. Electrophysiological studies and computational simulations suggest that βCD primarily modulates receptor desensitization and agonist binding/unbinding. While the overall trends in βCD-induced modulation of GABA-evoked currents were consistent across experiments, the magnitude of these effects varied significantly between individual cells. This variability may reflect differences in GABAa receptor subtypes, local receptor density, or the distinct microenvironment surrounding the receptors in different neurons.

Previous research on βCD effects at GABAa receptors is limited. For example, Shu et al. (2004) [129] reported that βCD influenced steroid-activated currents but did not alter responses elicited by GABA itself. Differences in experimental design, including the speed of agonist application and recording conditions, could explain this discrepancy. In addition, slow receptor-activating compounds may reveal modulation effects not apparent under rapid activation conditions, suggesting that βCD’s influence could be more detectable under specific kinetic contexts [130].

Further analyses indicate that βCD can differentially affect desensitization kinetics and the amplitude of currents in response to sub-saturating GABA concentrations. These observations suggest that βCD may interact with multiple functional domains of the receptor or influence receptor conformational flexibility in distinct ways. The precise molecular mechanism remains unclear, but it likely involves interactions with the receptor protein itself or its immediate lipid microenvironment, which can modulate the dynamics of receptor gating. Importantly, βCD-induced effects were reversible in all tested conditions, indicating a non-permanent modulation rather than receptor damage [130].

Overall, these findings highlight that β-cyclodextrin is more than a passive solubilizing agent. Its capacity to modulate receptor desensitization and agonist binding/unbinding could have implications for the design of cyclodextrin-based formulations for neuroactive compounds. Moreover, this modulation appears to depend on the specific receptor subtypes present and their local microenvironment, emphasizing the importance of considering cell type and experimental context when studying CD–receptor interactions.

As for the cholesterol, molecular dynamics simulations revealed its interactions with βCD, 2-hydroxypropyl-βCD (2HPβCD), and methylated βCD (MβCD). Cholesterol binding occurred fastest with MβCD, followed by βCD and 2HPβCD. Its polar oxygen remained exposed to water, favoring hydrophilic interactions. Binding altered CD structures, expanding their cavities, increasing circularity, and making 2HPβCD and MβCD more spherical. Van der Waals forces dominated the interaction, while Coulombic and hydrogen bonding contributions were minor. Functionalization of CDs enhanced van der Waals interactions and solubility. Free energy analyses indicated that 2HPβCD and MβCD were more flexible and could adopt multiple stable conformations, whereas βCD remained more rigid. Binding free energies confirmed that cholesterol association with all CDs is spontaneous and energetically favorable [131].

### 4.2. CDs with Neurotoxicity

As the growth of cEND cells is moderately sensitive to the presence of toxic compounds, the methodology applied allows clear discrimination between highly toxic, moderately toxic, and non-toxic samples. The results from this assay indicated that HP-β-CD did not exhibit any toxic effects on cEND cells, whether cultured in DMEM or in human serum, after incubation. Even under conditions where other CDs might show effects, HP-β-CD was well tolerated, confirming the previous literature reports [132].

For α-CD and TRIMEB, the assay revealed a notable difference in cell viability over time. Their cytotoxic effects were more pronounced in DMEM than in human serum, which appears to provide protective properties, likely due to interactions of serum proteins such as albumin with the CDs that reduce their bioavailability and toxicity. While luminescence measurements remained mostly above thresholds in lower exposure conditions, they were substantially reduced under conditions promoting higher cytotoxicity. TRIMEB consistently exhibited the highest level of cytotoxicity, whereas α-CD showed an intermediate effect.

Treatment with 10% DMSO, used as a positive control, produced a significant reduction in luminescence activity, confirming extensive cell death. Similar patterns have been reported in the literature regarding methylated cyclodextrin derivatives, where incorporation of hydroxypropyl groups mitigates cytotoxicity, whereas methyl substituents tend to increase it [133,134].

Taken together, the data confirm that toxic effects of CDs are generally greater in DMEM compared to heat-inactivated human serum, highlighting the protective role of serum components. Overall, the observed in vitro cytotoxicity follows the trend: TRIMEB as highly toxic, α-CD with moderate toxicity, and HP-β-CD as non-toxic, and dynamic toxicity profiles further support the relatively safe behavior of HP-β-CD compared to other CD derivatives [135].

### 4.3. Administration Routes for Cyclodextrin-Based Brain Delivery

The administration route plays a critical role in determining the efficiency of brain-targeted drug delivery. Among the available strategies, intravenous and intranasal administration are the most widely explored for cyclodextrin-based systems. Intravenous administration represents the conventional route, allowing systemic distribution and controlled dosing. In the context of cyclodextrin-based delivery, this route enables the use of functionalized nanocarriers designed to exploit receptor-mediated transcytosis or carrier-mediated transport across the blood–brain barrier. However, intravenous delivery is limited by rapid systemic clearance, potential off-target distribution, and the restrictive nature of the BBB, which may reduce effective brain exposure. In contrast, intranasal administration offers a non-invasive alternative that can partially bypass the BBB through direct nose-to-brain transport pathways, including the olfactory and trigeminal nerves. Cyclodextrins can enhance this route by improving drug solubility, stability, and mucosal permeability. Additionally, their ability to transiently modulate membrane properties may facilitate drug absorption across the nasal epithelium. Nevertheless, this approach is constrained by limited dosing volume, variability in absorption, and potential mucosal irritation upon repeated administration.

Overall, while intravenous delivery remains the most established route for controlled and targeted nanocarrier-based strategies, intranasal administration represents a promising complementary approach for enhancing brain delivery of cyclodextrin-based formulations, particularly for molecules with poor BBB permeability.

### 4.4. CDs in Alzheimer’s Disease (AD)

Alzheimer’s disease (AD) represents a chronic and progressively worsening neurodegenerative condition characterized by gradual deterioration of cognitive and functional abilities [136]. Its occurrence increases markedly with age, affecting a relatively small proportion of individuals in midlife while becoming significantly more prevalent in older populations [137]. A defining pathological feature is the accumulation of aberrant protein aggregates, particularly amyloid-β (Aβ), alongside a pronounced loss of cholinergic neurons. This neuronal decline highlights the critical role of acetylcholine in memory processing and higher brain functions [138]. As the disease advances, widespread neuronal damage and brain atrophy develop, ultimately leading to severe impairments in memory, behavior, and social functioning, making AD the leading cause of dementia.

Crocetin (CRT), a bioactive compound obtained from Gardenia jasminoides and Crocus sativus, has demonstrated multiple neuroprotective actions, including the reduction in neurotoxic species formation, interference with Aβ aggregation, destabilization of preformed fibrils, and facilitation of their clearance [139]. To improve its pharmaceutical applicability, Wong et al. incorporated CRT into a γ-cyclodextrin (γ-CD) delivery platform, resulting in markedly enhanced solubility and bioavailability [140]. The formulation exhibited very high encapsulation performance, supporting its feasibility for scalable production. Notably, the preparation avoided the use of organic solvents, minimizing potential toxicity concerns, and subsequent evaluations confirmed the safety of CRT, γ-CD, and their complex in both neuronal and AD-relevant cellular models.

In another approach, Park et al. developed reactive oxygen species-responsive nanoparticles designed for memantine delivery in AD treatment [141]. These nanosystems were constructed through conjugation strategies involving modified β-cyclodextrin and redox-sensitive linkers, followed by incorporation of memantine. Biodistribution analyses indicated a preferential accumulation within brain tissue, suggesting efficient targeting. Under oxidative stress conditions, neuronal cell models exhibited increased expression of NMDAR1 receptors; however, this effect was mitigated upon treatment with the memantine-containing cyclodextrin conjugates, indicating a protective mechanism at the receptor level.

Additionally, Sun et al. introduced a multifunctional nanosystem based on mesoporous nano-selenium combined with β-cyclodextrin nanovalves and borneol-mediated targeting [142]. This platform enabled controlled release of resveratrol in response to the oxidative microenvironment, facilitating transport across the blood–brain barrier and localized drug release. The system was shown to attenuate oxidative stress, reduce tau hyperphosphorylation, and protect neuronal cells, ultimately contributing to improved cognitive performance in experimental AD models. Furthermore, when compared to conventional administration of rivastigmine, cyclodextrin-based formulations demonstrated enhanced pharmacokinetic behavior, highlighting the advantages of such delivery strategies [143].

Collectively, these findings emphasize the versatility of cyclodextrin-based systems in addressing multiple pathological aspects of AD. By improving drug stability, enabling targeted delivery, and facilitating transport across the blood–brain barrier, these platforms provide valuable directions for the continued development of more effective therapeutic strategies in Alzheimer’s disease.

### 4.5. CDs in Parkinson’s Disease

Parkinson’s disease is a progressive neurodegenerative disorder primarily characterized by the degeneration of dopamine-producing neurons located in the substantia nigra region of the midbrain. This neuronal loss has been strongly linked to mitochondrial dysfunction, often associated with genetic factors, which contributes to cellular stress and eventual neuronal death [144]. A central pathological feature involves the accumulation of misfolded protein aggregates, predominantly composed of α-synuclein (α-syn), a protein that normally participates in the regulation of synaptic transmission under physiological conditions [145]. Disruptions in mitochondrial homeostasis are frequently accompanied by defects in autophagic processes, a mechanism also implicated in other neurodegenerative conditions such as Alzheimer’s disease [146].

Early investigations into the role of HPβCD in Parkinson’s disease employed human-derived neuroglioma cell models to replicate disease-relevant cellular changes. In these models, cells were engineered to express fluorescently labeled α-synuclein, allowing visualization of intracellular aggregation. Treatment with HPβCD led to a noticeable reduction in α-synuclein accumulation within cells. Further mechanistic analysis indicated that this effect was associated with the stimulation of autophagic pathways, promoting the intracellular clearance of protein aggregates. In particular, pathways involving lysosomal-associated membrane proteins, such as LAMP-2, were implicated in enhancing autophagosome formation and facilitating degradation processes [147]. This observation suggests a mechanistic overlap with pathways described in Niemann–Pick disease, where similar lysosomal systems are involved.

To better approximate the structural and functional complexity of brain tissue, more advanced in vitro models have been developed using three-dimensional midbrain organoids derived from pluripotent stem cells carrying Parkinson’s-associated mutations [145]. Within these systems, administration of HPβCD was associated with improved development and maintenance of dopaminergic neurons, indicating a potential restorative effect. Complementary in vivo studies using animal models further supported these findings, demonstrating that HPβCD treatment mitigated neuronal loss following induction of Parkinsonian pathology. Histological analyses confirmed preservation of dopaminergic neurons in treated subjects compared to untreated controls. However, it should be noted that these findings are primarily derived from in vitro and small animal studies, while evidence from large animal models and clinical validation remains limited and requires further investigation.

The growing body of evidence supporting the neuroprotective role of HPβCD has also led to the development of patented therapeutic approaches targeting dopaminergic neurodegeneration [148]. Collectively, these findings highlight the potential of cyclodextrin-based strategies to modulate key pathological processes in Parkinson’s disease, particularly through mechanisms involving protein aggregation and intracellular clearance pathways [149].

### 4.6. CDs in Glioblastoma

A study conducted by Turco et al. [150] explored the use of β-cyclodextrin-based nanoparticles (CDNPs) loaded with the Toll-like receptor 7/8 agonist R848 for targeting tumor-associated myeloid cells within GB. The findings demonstrated that CDNP-R848 exerts pronounced antitumor activity by converting the tumor microenvironment from an immunosuppressive to a pro-inflammatory state. Notably, this effect occurred independently of adaptive immune components such as T lymphocytes and natural killer cells, highlighting the central role of innate immune modulation, particularly through myeloid cell reprogramming.

The nanoparticles exhibited selective uptake by glioblastoma-associated macrophages and monocyte-derived myeloid populations within the tumor microenvironment, effectively overcoming the limitations typically imposed by the blood–brain barrier. This targeted internalization triggered a phenotypic shift in these cells, marked by increased expression of pro-inflammatory markers, including F4/80 and MHC-II, alongside elevated production of reactive oxygen species. Concurrently, a reduction in immunosuppressive myeloid-derived suppressor cell populations was observed, contributing to a less permissive environment for tumor progression. Mechanistically, activation of TLR7/8 signaling pathways was identified as a key driver of these changes, leading to enhanced secretion of pro-inflammatory cytokines such as IL-12 and TNF-α, which are essential mediators of immune activation. Despite the classical association of these cytokines with adaptive immune responses, the therapeutic effect in this context was primarily mediated by innate immune cells, particularly reprogrammed macrophages [151]. It should be noted that, in glioblastoma, many cyclodextrin-based nanocarriers primarily exploit regions of disrupted blood–brain barrier, which are characteristic of tumor-associated vasculature. However, in more infiltrative tumor areas where the BBB remains partially intact, effective delivery may depend on targeted strategies, such as receptor-mediated transcytosis, to facilitate transport across the endothelial barrier.

Preclinical evaluations further demonstrated that CDNP-R848 induced substantial tumor regression and improved survival outcomes, with some cases showing complete tumor elimination. Importantly, these therapeutic benefits were achieved with reduced systemic toxicity compared to free R848, which is often associated with widespread inflammatory side effects.

In a related approach, De La Torre et al. [152] developed AMC11, a β-cyclodextrin-based delivery system capable of forming supramolecular complexes with siRNA. These complexes provide protection against enzymatic degradation while facilitating efficient cellular uptake, enabling effective gene silencing. AMC11 showed high transfection efficiency across multiple tumor models, including glioblastoma, and successfully reduced the expression of key proteins involved in cell proliferation and survival pathways. Additionally, the system demonstrated low cytotoxicity, supporting its suitability for therapeutic applications.

Similarly, Manzanares et al. [153] introduced AMC6, another β-cyclodextrin-derived carrier optimized for siRNA delivery via macropinocytosis. This uptake mechanism, commonly active in tumor cells, enabled efficient internalization of the nanocomplexes. AMC6-based systems achieved strong gene silencing effects in glioblastoma models, significantly reducing the expression of proteins involved in cellular regulation. Although macropinocytosis was identified as the primary route of entry, inhibition of this pathway did not fully prevent uptake, indicating the involvement of additional mechanisms such as clathrin- and caveolin-mediated endocytosis. This redundancy in cellular entry pathways underscores the adaptability and effectiveness of AMC6 as a versatile delivery platform. Compared to lipid nanoparticles and viral vectors, cyclodextrin-based carriers generally exhibit lower transfection efficiency but offer important advantages in terms of biocompatibility, reduced immunogenicity, and improved safety profiles. While viral vectors provide high gene delivery efficiency, their clinical use is often limited by safety concerns, including immunogenicity and insertional mutagenesis. Lipid nanoparticles represent a well-established platform with high delivery efficiency; however, they may be associated with stability issues and off-target effects. In contrast, cyclodextrin-based systems provide a versatile and safer alternative, particularly suitable for applications requiring repeated administration, although further optimization is needed to enhance their delivery efficiency.

### 4.7. CDs in Niemann–Pick Disease

Hydroxypropyl-β-cyclodextrin (HPβCD) has attracted considerable attention as a potential therapeutic option for Niemann–Pick disease type C (NPD-C), particularly due to the lack of effective treatments. Its clinical relevance was acknowledged when it received orphan drug designation from regulatory agencies, including the U.S. Food and Drug Administration and the European Medicines Agency [154].

Initial clinical investigations focused on direct administration into the cerebrospinal fluid in very young patients, where treatment was associated with improved neuronal cholesterol homeostasis and a reduction in central nervous system pathology [155]. In early observational studies involving small patient groups, intrathecal delivery of HPβCD was linked to improvements in neurological functions such as cognition, swallowing, and motor coordination, although these findings were not supported by placebo-controlled comparisons [156]. Longer-term open-label studies also suggested that HPβCD may slow disease progression over time [157].

However, results from more rigorously designed clinical trials have been less conclusive. In a randomized, double-blind study comparing HPβCD treatment with a control procedure, no statistically significant differences were observed in primary clinical outcomes between groups [158,159]. In addition, treatment was associated with adverse effects, most notably hearing loss, which is consistent with earlier experimental evidence indicating ototoxicity related to HPβCD exposure [159].

These mixed findings ultimately led to the decision not to grant full regulatory approval for HPβCD in NPD-C at this stage [150]. Nevertheless, ongoing clinical efforts continue to investigate its therapeutic potential, with the aim of optimizing dosing strategies, improving safety profiles, and better defining its clinical benefits in affected patients [160].

### 4.8. CDs in Huntington’s Disease (HD)

HD is an inherited neurodegenerative disorder driven by the production of a mutant Huntingtin (HTT) protein, leading to progressive cognitive and motor decline [161]. In addition to genetic factors, disturbances in lipid homeostasis—particularly those involving intracellular transport mechanisms—have been implicated in disease progression [162,163].

Efforts to target the underlying molecular cause of HD have increasingly focused on gene-silencing strategies. In this context, Godinho et al. investigated the use of short interfering RNAs (siRNAs) deliveredusing modified amphiphilic β-cyclodextrins [164]. These carriers formed stable nanoscale complexes under physiological conditions and enabled efficient reduction in HTT expression in both neuronal cell models and patient-derived fibroblasts, while maintaining low cytotoxicity. In vivo experiments further demonstrated that localized administration of CD–siRNA systems could induce sustained gene silencing and improve motor function in HD mouse models.

Complementary work by Mendonça et al. examined chemically tailored cyclodextrins as delivery platforms for antisense oligonucleotides targeting HTT [165]. Among the tested systems, modified γ-cyclodextrins showed enhanced cellular uptake and effectively reduced mutant protein levels. Functionalization with the rabies virus glycoprotein peptide further improved targeting efficiency, resulting in a more pronounced suppression of disease-related effects.

Beyond nucleic acid delivery, cyclodextrins have also been explored as solubilizing agents for small-molecule therapeutics. For instance, suberoylanilide hydroxamic acid, a histone deacetylase inhibitor, demonstrated improved brain availability and therapeutic efficacy when administered as a CD complex. This formulation enabled systemic delivery while enhancing pharmacological activity, leading to measurable improvements in motor performance in HD models [166].

Further advancements combined targeting and delivery strategies by incorporating brain-penetrating peptides into CD-based nanocarriers loaded with siRNA [167]. These systems were shown to cross in vitro blood–brain barrier models, release their cargo neuronal cells, and induce effective downregulation of HTT expression. Collectively, these findings highlight the versatility of cyclodextrins as multifunctional carriers for both gene-based and small-molecule therapies in HD, supporting their broader potential in central nervous system drug delivery.

### 4.9. CDs in Epilepsy

Electrophysiological studies have demonstrated that β-CD can be used to solubilize pharmacological agents such as rufinamide (RUF) for application in hippocampal slices, providing insights into seizure-like events (SLEs) modulation. Analysis of SLE dynamics revealed that β-CD-solubilized RUF prolonged the preictal phase while simultaneously reducing the duration of both ictal and postictal phases, suggesting a shortening of the active seizure period and faster post-seizure recovery [168]. The absolute frequency of SLEs was observed to increase under RUF treatment, yet detailed examination of ictal spike firing patterns indicated that the first set of spikes remained largely unchanged, whereas the second set exhibited elevated firing rates compared to controls. In contrast, control SLEs displayed a notable deceleration in spike firing during the same period. These findings imply that RUF, when delivered via β-CD, maintains consistent firing activity during ictal events and promotes stabilization of neuronal membranes after seizures. Notably, no significant alterations were observed in tPAC Max across any analyzed phase or frequency range, reinforcing the notion that β-CD-assisted RUF administration primarily affects seizure duration and recovery dynamics without broadly disrupting network oscillatory patterns [168]. Overall, this evidence supports the utility of β-CD as a drug delivery platform to enhance solubility while preserving or modulating electrophysiological properties, with implications for improving seizure management and minimizing neuronal damage [169].

### 4.10. CDs in Multiple Sclerosis (MS)

In contrast to the conventional strategy, where an excess of CD is typically employed to improve drug solubility under the assumption that such an approach also protects acid-sensitive compounds from degradation in the gastric environment, the objective in this case was to formulate a saturated complex without surplus CD. This approach was guided by evidence suggesting that an excess of CD can negatively influence the absorption of cladribine from both solid oral dosage forms and transmucosal delivery systems [170,171]. It was hypothesized that maintaining the drug in a saturated complex would preserve cladribine at its maximum thermodynamic activity when it comes into contact with mucosal tissues, thereby potentially improving its therapeutic performance.

Following this rationale, a formulation based on a distinctive dual cladribine–HPβCD complex was created. This system represents an amorphous mixture composed of two different types of interactions: an inclusion complex, in which the hydrophobic portion of the cladribine molecule is accommodated within the cavity of the cyclodextrin, and a non-inclusion complex, which arises from hydrogen bonding between amorphous cladribine molecules and the external hydroxyl groups present on the cyclodextrin structure [170]. The preparation process was optimized through carefully controlled conditions, including elevated temperatures, extended complexation periods, and subsequent lyophilization, in order to maximize drug incorporation while preserving the amorphous nature of the final product.

The formation of this dual complex is enabled by the relatively hydrophilic character of cladribine, which exhibits moderate aqueous solubility (approximately 4.5–5 mg/mL) [172], combined with the amorphous properties of partially substituted HPβCD. These characteristics facilitate the generation of a supersaturated system that, upon cooling, retains an excess amount of cladribine in a non-crystalline state. In such systems, it is generally observed that around 60–70% of the drug is present in the non-inclusion form, while the remaining fraction exists as a conventional inclusion complex. Importantly, this ratio can be modulated by adjusting both the cyclodextrin concentration and the processing parameters [53]. Overall, this dual-complex configuration significantly enhances drug solubility and enables the successful formulation of cladribine into oral tablet dosage forms [173].

A comparable application of cyclodextrins can be observed in the development of chitosan-based nasal powders containing dimethyl fumarate–cyclodextrin binary systems for the treatment of multiple sclerosis. In this context, cyclodextrins have been shown to improve the chemical stability of DMF. Specifically, the formation of binary complexes reduces the susceptibility of DMF to hydrolytic degradation into monomethyl fumarate. Among the investigated systems, the DMF–RAMEB complex at a 1:2 molar ratio demonstrated the most favorable stability profile. This finding is particularly relevant given that randomly methylated β-cyclodextrin (RAMEB) is widely regarded in the literature as a suitable cyclodextrin derivative for nasal drug delivery applications [174].

The interaction between DMF and RAMEB was further substantiated through multiple analytical techniques, including thermal analysis, Fourier-transform infrared spectroscopy, and powder X-ray diffraction, all of which confirmed successful complex formation. Subsequent investigations focused on comparing various preparation techniques commonly employed for producing nasal powders containing API–CD complexes. To assess the stabilizing effect of complexation, a freeze-drying study was conducted using a Design of Experiments approach. The DMF–RAMEB complex with a 1:2 molar ratio again demonstrated superior stabilization and was therefore selected for the development of a dry, mucoadhesive nasal powder formulation [174].

Overall, the combination of cyclodextrin complexation and freeze-drying technology represents an effective strategy to address the inherent limitations of DMF, particularly its instability and limited aqueous solubility. This approach highlights the broader potential of cyclodextrin-based systems in improving the delivery and performance of drugs used in the management of multiple sclerosis.

The diversity of cyclodextrin-based platforms, their mechanisms of transport across the blood–brain barrier and their therapeutic outcomes in central nervous system disorders are comparatively summarized in Table 3.

As shown in Table 3, the most promising strategies are based on functionalized cyclodextrin nanocarriers that combine targeting ligands with controlled drug release and disease-specific therapeutic cargo. Cyclodextrin-based nanocarriers should be considered within the broader landscape of brain delivery platforms, including lipid nanoparticles, polymeric nanoparticles, exosomes, and viral vectors. Compared to these systems, cyclodextrins offer distinct advantages related to their well-established safety profile, low immunogenicity, and high formulation versatility, particularly through inclusion complex formation and chemical functionalization. However, their intrinsic ability to cross the blood–brain barrier is limited, and efficient delivery often requires incorporation into nanostructured or ligand-targeted systems. In contrast, viral vectors and certain lipid-based platforms exhibit higher delivery efficiency but are associated with safety concerns, including immunogenicity, off-target effects, and limited suitability for repeated administration. Overall, cyclodextrin-based systems represent a flexible and biocompatible alternative, with strong potential for integration into multifunctional delivery platforms, although further optimization is required to achieve comparable delivery efficiency.

## 5. Methods

A structured literature search strategy was applied, including predefined databases, keywords, and inclusion/exclusion criteria. This review was designed as a narrative synthesis incorporating selected elements of a structured literature search, guided by key principles of the PRISMA (Preferred Reporting Items for Systematic Reviews and Meta-Analyses) framework. A comprehensive search was conducted using Google Scholar, PubMed, and ScienceDirect databases, applying combinations of keywords such as “cyclodextrins”, “blood–brain barrier”, “BBB transport”, “drug delivery”, “ADMET”, “solubility”, “stability”, “inclusion complexes”, “cyclodextrin polymers”, “endocytosis”, “neuroreceptors”, “GABA receptors”, “neurotoxicity”, “Alzheimer’s disease”, “Parkinson’s disease”, “Niemann–Pick disease”, “multiple sclerosis”, “amyotrophic lateral sclerosis”, “Huntington’s disease”, and “glioblastoma”. Emphasis was placed on the literature published within the past five years, while earlier studies were incorporated where they provided essential mechanistic or foundational insights.

Eligible studies were selected based on their relevance to at least one of the following domains: (i) structural and functional characteristics of the blood–brain barrier, including mechanisms governing selective permeability and targeted delivery strategies; (ii) physicochemical and pharmacokinetic properties of cyclodextrins, with particular attention to ADMET-related considerations; (iii) the influence of cyclodextrins on drug solubility, stability, and inclusion complex formation, including interactions with natural compounds; (iv) the design and functional behavior of cyclodextrin-based polymers; (v) cellular uptake pathways, especially endocytic mechanisms associated with cyclodextrins; and (vi) molecular and biological interactions of cyclodextrins with neural components, including receptor modulation, membrane-associated processes, neurotoxicity profiles, and their implications across a spectrum of neurological and oncological conditions.

**Figure 5:**
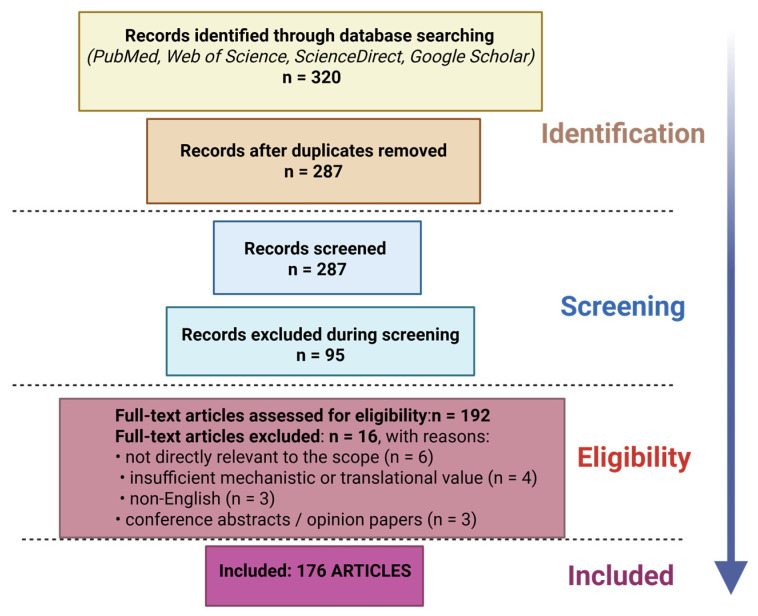
PRISMA-ScR-informed flow diagram illustrating the study identification, screening, and inclusion process. Created in BioRender. Varut, M. (2026); https://BioRender.com/071ce7p (accessed on 2 February 2026).

Only full-text articles published in English were included, while abstracts, opinion pieces, and studies lacking direct relevance to the scope of this review were excluded. The objective of this work is to integrate current knowledge on cyclodextrins within the context of blood–brain barrier dynamics, highlighting mechanistic links between their physicochemical properties and biological performance, rather than to perform a quantitative meta-analysis or formal assessment of study bias (Figure 5).

## 6. Future Perspectives

Looking ahead, advancing our understanding of how various CDs and their chemical derivatives engage with neuronal targets, receptors, and transport pathways will be critical. In particular, the precise mechanisms by which CD-based inclusion complexes release drugs or bioactive molecules across the BBB remain incompletely defined. Unraveling these interactions could inform the design of more efficient CD formulations with enhanced central nervous system bioavailability, improved pharmacokinetics, and minimized off-target effects. Future studies may further explore the potential of cyclodextrins (CDs) in addressing complex neurodegenerative disorders such as Alzheimer’s, Parkinson’s, Huntington’s disease, and Niemann–Pick disease. Optimization of CD properties—including cavity size, chemical functionalization, and polymeric architecture—represents a key strategy for enhancing selective drug delivery, improving cellular uptake, and minimizing neurotoxicity. In particular, chemical modifications such as hydroxypropylation and methylation have been shown to improve aqueous solubility, membrane interaction, and cholesterol affinity, while the degree of substitution critically influences pharmacokinetic behavior and safety profiles. In addition, functionalization with targeting ligands, including peptides, transferrin, or folate, enables receptor-mediated transcytosis across the blood–brain barrier, whereas cyclodextrin-based polymers, such as polyrotaxanes and grafted systems, provide improved stability, controlled drug release, and enhanced transport properties. The integration of these approaches with nanoparticle platforms and stimuli-responsive systems may facilitate the development of next-generation brain-targeted therapies with improved efficiency and reduced systemic exposure. Finally, the interplay between CDs, neural health, and systemic physiology warrants further investigation. Understanding how CD-mediated modulation of oxidative stress, protein aggregation, and lipid microenvironments influences disease progression may open new avenues for preventive and adjunctive therapies. Overall, combining mechanistic insights with advanced cyclodextrin design holds significant promise for expanding the therapeutic landscape of central nervous system disorders.

## 7. Limitations

Despite adopting a structured search approach across multiple databases and drawing on elements of PRISMA guidelines, this review was performed as a narrative synthesis rather than a comprehensive systematic review. Consequently, the total number of studies screened, assessed for eligibility, or excluded, along with specific reasons for exclusion, were not formally documented.

Selection bias may have influenced the included studies, as decisions on eligibility were guided by subjective judgment. Furthermore, the absence of quantitative meta-analysis limits the capacity to directly compare the effects of different cyclodextrin types, derivatives, or formulations on neuroprotective mechanisms, drug delivery efficiency, and modulation of pathological processes in various neurological disorders.

To strengthen the evidence base, future investigations should incorporate systematic review methodologies with transparent study selection criteria and, where feasible, quantitative synthesis. Such analyses would provide a more robust framework for assessing the relative performance of CDs across diverse neurodegenerative and neuroinflammatory contexts. Although cyclodextrins are generally regarded as safe and well-tolerated excipients, their toxicity profile is influenced by chemical structure, degree of substitution, and administered dose. Certain derivatives, particularly methylated cyclodextrins, may induce membrane disruption through cholesterol extraction, leading to cytotoxic effects at higher concentrations. In addition, while cyclodextrins exhibit low intrinsic immunogenicity, functionalized or nanoparticle-based systems may trigger immune responses depending on surface modifications and targeting ligands. These considerations are particularly relevant for repeated or long-term administration in central nervous system disorders. Therefore, careful optimization of cyclodextrin type, formulation design, and dosing strategies is essential to balance therapeutic efficacy with safety and tolerability.

## 8. Conclusions

Cyclodextrins have emerged as multifunctional carriers with significant potential in the treatment of neurological disorders. Beyond enhancing drug solubility, they facilitate targeted delivery across the blood–brain barrier, improve pharmacokinetics, and mitigate neurotoxicity, making them applicable to Alzheimer’s, Parkinson’s, Niemann–Pick, and Huntington’s diseases, multiple sclerosis, and glioblastoma.

Preclinical and early clinical studies demonstrate that CD-based systems can stabilize drugs, enable receptor- or endocytosis-mediated delivery, and support innovative therapies such as siRNA or nanoparticle platforms. These strategies highlight the versatility of CDs for neuroprotection, precision targeting, and modulation of disease pathways. It is important to emphasize that the therapeutic mechanisms of cyclodextrin-based systems differ fundamentally between neurodegenerative disorders and brain tumors. In neurodegenerative diseases, such as Alzheimer’s or Parkinson’s diseases, cyclodextrins are primarily used to support long-term neuronal protection by modulating lipid homeostasis, reducing protein aggregation, and restoring cellular function. In contrast, in brain tumors such as glioblastoma, cyclodextrin-based platforms are designed to enhance drug delivery across the blood–brain barrier and promote cytotoxic effects within tumor cells. These distinct therapeutic objectives require different design strategies, dosing regimens, and safety considerations. Therefore, future development of cyclodextrin-based systems should carefully account for this dichotomy in order to optimize clinical outcomes across diverse central nervous system pathologies. In addition to scientific and technological challenges, the clinical translation of cyclodextrin-based delivery systems is also influenced by regulatory considerations. Complex formulations combining cyclodextrin polymers, targeting ligands, and active pharmaceutical ingredients present significant challenges for regulatory approval by agencies such as the FDA and EMA. These multi-component systems require comprehensive characterization of their physicochemical properties, stability, and reproducibility, as well as detailed evaluation of pharmacokinetics, toxicity, and potential immunogenicity. Furthermore, the interplay between individual components may complicate standard approval pathways, necessitating tailored regulatory strategies. Addressing these challenges will be essential for the successful translation of advanced cyclodextrin-based platforms from preclinical research to clinical application.

Future research should prioritize translational studies and clinical trials to identify optimal CD derivatives, formulations, and dosing strategies for specific neurological conditions. By bridging mechanistic insights with clinical applications, CDs hold promise as a cornerstone of next-generation neurotherapeutics, offering safer and more effective interventions for CNS disorders.

## Figures and Tables

**Table 1:** Transporter-mediated transportation through BBB.

Transporter	Abbreviation/Gene	Expression at BBB	Function/Role in Brain Delivery	Delivery Transport Characteristics/Limitations	Ref.
Glucose Transporter	GLUT1/SLC2A1	Highly expressed in brain endothelial cells	Facilitates transport of glucose into the brain; can be exploited for dual-targeted strategies across intestinal and BBBs	Enables delivery of glucose-mimetic drugs and prodrugs; widely explored for carrier-mediated transport strategies	[41]
L-type Amino Acid Transporter	LAT/LAT1	Highly expressed at BBB and tumor cells	Mediates uptake of large neutral amino acids; enables enhanced CNS drug delivery and may improve therapeutic outcomes in neurological disorders	One of the most exploited transporters for BBB drug delivery due to high expression and substrate specificity	[42,43]
Glutathione Transporter	GSH transporter	Enriched at BBB	Transports glutathione into the brain; supports antioxidant delivery and has been applied in models of autoimmune and neurodegenerative diseases	Used in glutathione-targeted delivery systems to enhance brain uptake of conjugated drugs	[44,45]
Efflux Transporters	P-gp (ABCB1), BCRP (ABCG2), MRP	Highly expressed at BBB endothelial cells	Actively pump out xenobiotics and therapeutic agents, maintaining selective permeability but limiting brain drug accumulation	Major barrier to CNS drug delivery; key targets for inhibition or circumvention strategies	[46,47]
Organic Anion Transporting Polypeptides	OATP/OATP1A2, OATP1A4	Expressed on BBB and choroid plexus epithelial cells	Facilitate uptake of endogenous and exogenous organic anions; involved in clearance from cerebrospinal fluid and delivery of neuroprotective agents (e.g., statins)	Potential pathway for delivery of specific drugs; role still under investigation	[48,49,50]
Monocarboxylic Acid Transporters	MCT/MCT1	Present at BBB	Transport monocarboxylic acids such as lactate, pyruvate, ketone bodies, and short-chain fatty acids; provide a pathway for energy metabolites and therapeutic molecules	Can be exploited for delivery of metabolite-like drugs and prodrugs targeting energy pathways	[51]

**Table 2:** Tight junction proteins.

Tight Junction Protein	Type/Localization	Role at BBB	Relevance for CNS Drug Delivery	Ref.
Claudins (e.g., Claudin-5)	Transmembrane protein in BMECs	Major structural component of intercellular tight junctions; regulates BBB permeability and integrity	Key regulators of BBB tightness; modulation may enhance drug permeability across the barrier	[52,53]
Occludin	Transmembrane protein in BMECs	Facilitates homophilic junction formation; modulates paracellular permeability	Involved in dynamic regulation of BBB permeability; potential target for transient barrier opening	[52,53]
Junctional Adhesion Molecules (JAMs, e.g., JAM-A)	Adhesion molecules in BMECs	Modulate paracellular transport and leukocyte infiltration via tight junction formation	May influence immune cell trafficking and drug passage under pathological conditions	[52,54]
Zonula Occludens (ZO-1, ZO-2, ZO-3)	Cytoplasmic scaffolding proteins	Anchor tight junction transmembrane proteins to the cytoskeleton; maintain junctional stability; ZO-1 ablation disrupts BBB integrity	Indirect regulators of BBB integrity; disruption may increase permeability but with safety concerns	[52,55]

**Table 3:** Comparative overview of cyclodextrin-based delivery systems for brain targeting, highlighting the transport mechanisms across the blood–brain barrier, therapeutic applications and current translational limitations.

Cyclodextrin System	Therapeutic Cargo	Target Disease	BBB Transport Mechanism	Experimental Model	Main Therapeutic Outcome	Limitations	Development Stage
HP-β-CD	Cholesterol mobilization	Niemann–Pick type C	Endocytosis/membrane cholesterol extraction	In vivo/clinical	Reduced lysosomal cholesterol accumulation, neuroprotection	Ototoxicity, intrathecal administration required	Clinical [154,155,156,157,158,159]
β-CD inclusion complexes	Poorly soluble neuroactive drugs	Alzheimer’s disease	Increased solubility → enhanced endothelial uptake	In vitro/in vivo	Reduced Aβ aggregation, improved brain exposure	Limited targeting specificity	Preclinical [140,141,142,143]
Functionalized CD nanoparticles	Anti-amyloid drugs	Alzheimer’s disease	Receptor-mediated transcytosis	In vitro BBB models/in vivo	Increased brain delivery and controlled release	Complex formulation design	Preclinical [141,142,143]
HP-β-CD	Autophagy modulators	Parkinson’s disease	Endocytosis/intracellular trafficking modulation	In vitro/in vivo	Enhanced autophagic clearance and neuroprotection	Systemic toxicity at high doses	Preclinical [146,147,148,149]
CD-based nanocarriers	siRNA/chemotherapeutics	Glioblastoma	Receptor-mediated transcytosis/adsorptive transcytosis	In vitro BBB/orthotopic models	Gene silencing, tumor growth inhibition	Tumor heterogeneity, limited penetration depth	Advanced preclinical [150,151,152]
Polyrotaxane–CD systems	Small-molecule drugs	Neurodegenerative disorders	Sustained release + endocytosis	In vitro/in vivo	Prolonged circulation time, improved cellular uptake	Limited clinical data	Preclinical [93,94,95,96]
CD–polymer conjugates	Hydrophobic anticancer drugs	Brain tumors	Enhanced permeability + vesicular transport	In vivo	Increased drug stability and brain accumulation	Scale-up challenges	Preclinical [100,101]
Targeted CD nanoplatforms (TfR/LRP1 ligands)	Multiple cargos	CNS disorders	Receptor-mediated transcytosis	In vitro BBB/in vivo	High brain targeting efficiency	Ligand immunogenicity	Preclinical [8,30,32]

## Data Availability

No new data were created or analyzed in this study. Data sharing is not applicable.
